# A survey of train driver schedules, sleep, wellbeing, and driving performance in Australia and New Zealand

**DOI:** 10.1038/s41598-022-07627-0

**Published:** 2022-03-10

**Authors:** Jillian Dorrian, Janine Chapman, Lorelle Bowditch, Nora Balfe, Anjum Naweed

**Affiliations:** 1 University of South Australia, UniSA Justice and Society, Adelaide, 5001 Australia; 2 Flinders University, Adelaide, Australia; 3 Central Queensland University, Appleton Institute for Behavioural Science, Wayville, SA 5034 Australia; 4 Trinity College Dublin, Centre for Innovative Human Systems, Dublin, Ireland

**Keywords:** Psychology, Human behaviour

## Abstract

Train drivers work long hours on 24 h schedules and many factors impact their fatigue risk at work, creating a clear imperative for good rostering practice. Adopting a systems approach, this study investigated the relationship between multiple interrelated factors (train drivers’ schedule, sleep, wellbeing, and fatigue) and the perceived influence of these factors on train driving performance and safety using an online survey distributed in Australia and New Zealand. In addition to demographics and work schedule, passenger and freight train drivers (*n* = 751) answered questions about: (1) Sleep duration; (2) wellbeing, including physical and mental health, the extent to which shiftwork causes sleep, social, domestic, and work problems, and (3) the extent to which ten fatigue, health and wellbeing factors in the work and home environment negatively impact their driving performance. The key factor that emerged from analyses, with the largest and most consistent negative effects (and controlling for other factors) was schedule irregularity. Approved rosters were ranked as having the most important impact on day-to-day driving performance, followed by physical and mental health, and outside work factors. Results also suggested that schedule irregularity may amplify the negative impacts of the roster, impaired physical and mental health, and outside work factors on driving performance. As shift variability and schedule irregularity are often poorly represented in existing industry guidance, these results provide evidence for increased reflection on current fatigue management guidelines for train drivers and suggest a need for greater focus on schedule irregularity through the lens of a systems approach.

## Introduction

The work of a train driver is demanding, requiring the prolonged exertion of mental effort and concentration in often monotonous, yet highly time pressured, environments^1–5^. This combination of sustained vigilance, task demands and pressure represents a high risk for fatigue^2,6^ particularly as drivers work long hours on 24 h schedules. This contributes to fatigue risk by negatively impacting hours of restorative sleep^7^, extended periods of wakefulness and disrupting circadian rhythms^8,9^, and can lead to further difficulties such as falling asleep and drowsiness at work^10^. Shift schedules in rail are also frequently irregular and unpredictable, including early morning and night shifts with relatively short between-shift intervals. These factors are reported to further exacerbate sleep and circadian rhythm issues in drivers^11,12^.

The issues associated with shift-work induced fatigue ultimately impact driving performance and safety^4,13,14^. For example, high workload and high levels of driver fatigue have been linked to cognitive disengagement from the driving task, leading to an increased accident risk^14^. Similarly, a range of studies have demonstrated performance deficits and impairment in relation to lowered alertness and deterioration in attention, reduced reaction time, poor decision making, and risk of microsleeping at work^11,15,16^. Fatigue and performance issues may also be differentially impacted depending on the rail mode. For example, passenger systems, typically characterised by shorter travel distances and single driver operations, are considered more tactical in that driving requires enhanced diagnosis and error detection skills^17^. In contrast, freight systems typically travel much further distances (especially in countries such as Australia and New Zealand, where population densities are much lower than in the USA or Europe), feature differences in roster design, and can involve two-driver/relay operations^3^. The elevated requirements for vigilance and attention under such conditions are likely to render the train driving task more sensitive to the negative impacts of fatigue^2,4,6,14^. In the US, freight drivers are found to have the highest fatigue risk due to more unpredictable and night shifts and longer hours^18^.

In addition to fatigue and scheduling, evidence from the transport literature shows a number of other factors that can impact negatively on driver performance and safety. These include physical health and mental health factors including substance use^19–21^. Another important but rarely considered factor that may be associated with driver performance is work-life balance and home-work commute time. Qualitative research in Australian rail^22–24^ suggests that outside work factors play a large role in the mental health and fatigue of drivers, and interviews from the UK suggest that rail employees consider these factors to be associated with safety risk^25^.

As well as having a direct impact on driving performance and safety, it is likely that the factors outlined above may also have an indirect impact through their interaction with other factors. For example, Chapman et al.^26^ published novel data showing that the risk posed to safety through train drivers’ physical health may be mitigated by compensatory non-workday sleep. However, most studies to date have tended to focus on factors separately, rather than examining their relative impacts. This is important to gain a comprehensive picture of the range of contributory influences, consistent with a systems framework^27^. Furthermore, while a large body of research has been directed towards impacts on driving performance in passenger transport generally, there has been less research effort directed at rail^28,29^, representing a significant gap in the literature.

This paper therefore takes a systems perspective by examining driver scheduling, fatigue, and multiple other potential contributing and interrelated factors, to assess their impact on driving performance and safety. The data are drawn from a large representative survey of train drivers in Australia and New Zealand, gaining the perspectives of the drivers directly, which is aligned with current practice in the rail safety literature^22,23,30^. Investigating these issues in context with one another, with critical driver input, will facilitate targeted interventions to improve rail driver performance and safety in the areas they are most needed.

In light of the above, the research aims of the current paper were twofold: First, to examine the relationship between train drivers’ schedules and sleep, wellbeing, and fatigue; and second, to determine the extent to which these factors are perceived to influence train driving performance and safety.

## Methods

The online survey was accessed by 1146 and completed by 751 (66%) train drivers, employed in Australia or New Zealand. The survey was co-designed with a national Australasian rail safety group^31^, including more than twenty rail organisations. Group members and their networks supported survey piloting (*n* = 31) and administration. Participants were recruited through employing organisations and online (Facebook, reddit, and Railpage.com.au). This study was approved by the Central Queensland University Human Research Ethics Committee (Approval no. 0000021339). All research was performed in accordance with relevant guidelines and regulations, we only recruited adults (18+ years), and in responding to the anonymous survey, participants indicated their informed consent.

### Measures

In addition to demographics, age (in years) and gender (1 = male, 2 = female, 3 = other, 4 = prefer not to say), and a series of questions relating to signals passed at danger (SPADs—an incident where the train exceeds the limit of its movement authority and attracts collision risk)^32,33^, respondents completed questions relating to work, sleep, wellbeing, and the impact of these factors on their driving performance.

#### Work

Participants responded to questions relating to driving experience (years), days worked per week (days), shift length (hours), rail mode (1 = freight, 2 = passenger), and shift pattern (1 = permanent day or evening; 2 = permanent night or other including night; 3 = day and evening; 4 = day and night; 5 = day, evening, and night). Participants also responded to a question relating to the degree of perceived pattern regularity, “In general, how regular, or irregular, is your shift system?” (1 = very regular or regular; 2 = sometimes regular, sometimes irregular; 3 = irregular; 4 = very irregular), which was an adapted, simplified question from the Standard Shiftwork Index (SSI)^34^. The descriptions of these concepts from the SSI state that a regular shift system may be “a fixed roster which is repeated when the cycle of shifts finishes, even if occasional variations occur to meet special requests” (SSI, question 1.29^34^). This compares to an irregular roster, where “the duty roster does not cycle or repeat in any regular manner and individual preferences are not taken into account” (SSI, question 1.29^34^).

#### Sleep

Questions included amount of sleep per 24 h period (hours) and sleep quality (10-point scale, higher = better quality) on workdays and days off, and ratings of satisfaction (“How do you feel about the amount of sleep you usually get?” from the Standard Shiftwork Index^34^, 1 = nowhere near enough; 2 = could do with a lot more; 3 = could do with a bit more; 4 = get the right amount; 5 = get plenty).

#### Wellbeing

Participants rated their frequency of tiredness in the past 12 months (1 = almost never; 2 = sometimes; 3 = rarely; 4 = frequently; 5 = almost always, adapted from the Standard Shiftwork Index^34^), and rated symptoms of psychological wellbeing on an adapted four-item version of the Patient Health Questionnaire (PHQ-4)^35^. Questions asked how frequently the respondent was bothered by a series of symptoms (e.g. “little interest or pleasure in doing things,” 1 = never, 2 = rarely, 3 = sometimes, 4 = often, 5 = very often) associated with mental wellbeing. Internal consistency for the PHQ-4 was high in this dataset ($\alpha =0.88$), with inter-item correlations ranging from *r* = 0.55 to *r* = 0.72.

#### Factors impacting on train driving

The perceived extent to which a series of factors negatively impacted respondents' personal driving performance was assessed using an adapted version^32^ of the 56-item scale developed from the RSSB incident factor framework^36^. There were ten items relating to individual fatigue, health and wellbeing. Respondents were asked, “To what extent do factors related to fatigue, health and wellbeing negatively impact your driving performance, on a day-to-day basis?” Items included physical health, mental or emotional health, medication (prescribed or over the counter), other substances (e.g. alcohol, recreational or illegal drugs), not complying with medical requirements or treatments (e.g. not wearing glasses when required, not attending medicals), a shift/roster pattern approved by the organisation contributing to fatigue (e.g. long hours, inadequate or poorly timed rest breaks, difficulty getting enough sleep due to shift pattern), a shift/roster pattern which was NOT approved by the organisation contributing to fatigue (e.g. unofficial shift swap), commute (long journey to/from work), factors outside of work contributing to fatigue (e.g. sleepy because of noisy neighbours affecting sleep), other well-being issues (e.g. unhealthy lifestyle, thirst, hunger). Participants responded to each item on a 5-point scale (1 = no extent, 2 = little extent, 3 = some extent, 4 = moderate extent, 5 = great extent). Internal consistency for the ten fatigue, health, and wellbeing items was high in this dataset ($\alpha =0.92$), with inter-item correlations ranging from *r* = 0.30 to *r* = 0.80.

### Data processing and analysis

Analyses were conducted using STATA 15.0 (StataCorp TX, 2017) and jamovi 1.2 (The jamovi project, 2020). Since only 1% of the sample responded to the gender question as “other” or “prefer not to say,” gender is only represented by a binary variable (male/female) in the analyses presented. Independent samples t-tests were used to compare age and driving experience between rail modes (passenger/freight) and to compare sleep duration and quality on workdays compared to days off. A series of models specified covariates of age (years), gender (male/female), days worked per week (days), shift length (hours), rail mode (freight/passenger), regularity rating (regular/sometimes/irregular/very irregular), and shift pattern (permanent day or evening/permanent night or other including night/day and evening/day and night/day, evening, and night). Specifically, (a) analysis of covariance (ANCOVA) models specified hours of sleep per 24 h and sleep quality ratings on workdays and days off as dependent variables; (b) ordinal regression models specified sleep satisfaction and tiredness ratings as dependent variables; (c) ANCOVA specified average PHQ-4 score as a dependent variable; and (d) multivariate analysis of covariance (MANCOVA) models specified the perceived impact on driving performance scores for the ten fatigue, health, and wellbeing factors, as dependent variables. Post-model ANOVA with Tukey HSD post-hocs were conducted for the significant covariates identified in the MANCOVA.

## Results

### Sample description

Broadly representative of the average profile of train drivers in Australia^37^, the mean age of respondents was 46.2 (± 10.8) years and the majority of drivers were male (87%), with 12% female, and 1% reporting as other, or indicating that they prefer not to say. The majority worked in Australia (88%), and the remainder in New Zealand, with 17.3 (± 14.9) years driving experience, working 5.4 (± 0.9) days per week. Drivers were from freight (47%) and passenger (53%) modes. Most freight drivers worked in heavy haul (67%, coal, iron ore) and 30% in intermodal environments. The majority of passenger drivers worked in urban environments (11% regional). While drivers in freight and passenger rail modes had equivalent distributions for gender and country of work, there were other demographic differences. Freight drivers were older (48.7 ± 0.6 years) than passenger drivers (44.0 ± 0.5 years, $t_{749}=6.1, \textit{p}<0.001$), and had a longer history of driving experience (freight = 19.8 ± 14.6 years, passenger = 15.0 ± 14.8 years, $t_{749}=4.4, p<0.001$).

**Figure 1:**
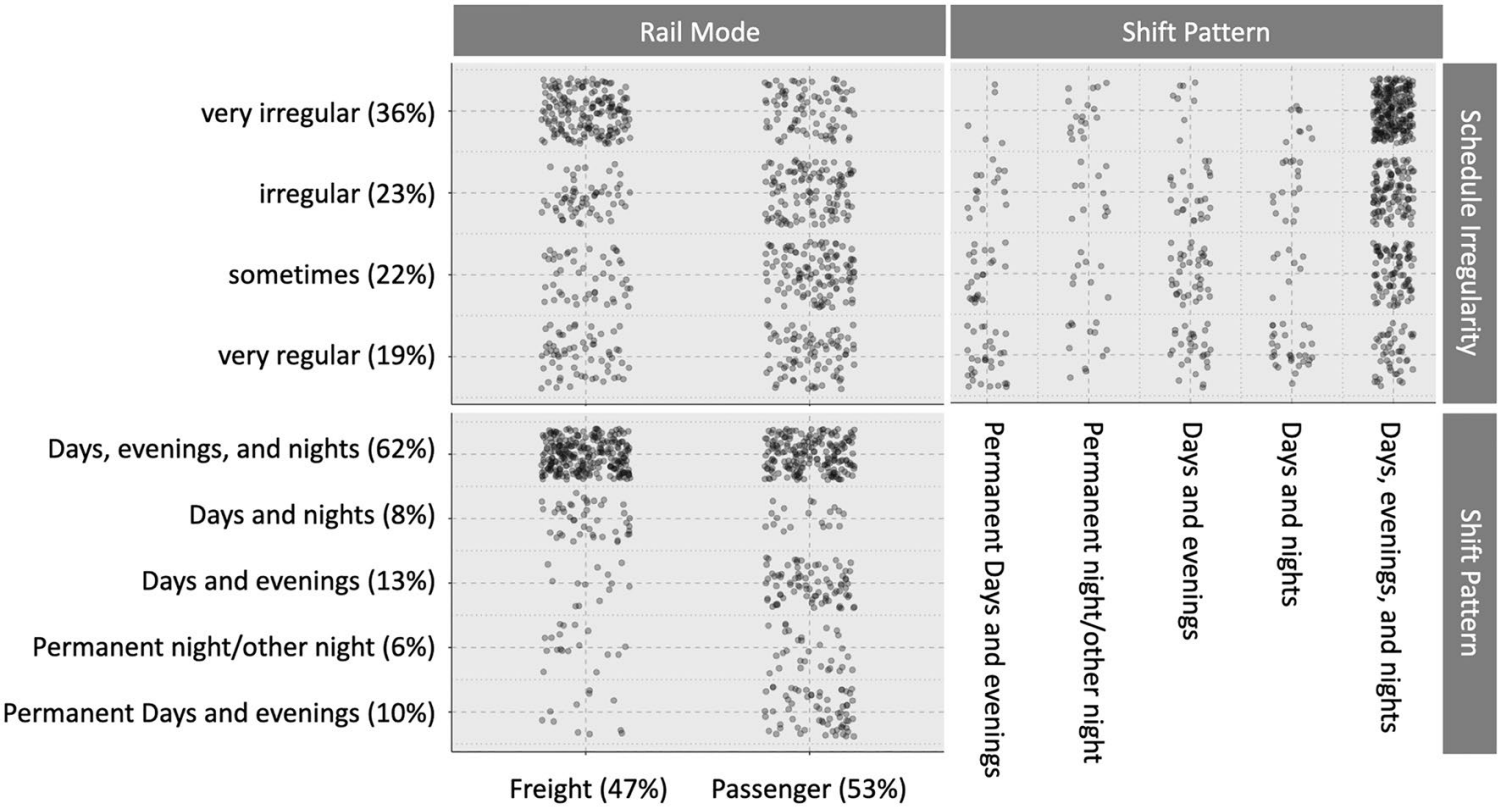
Jitter matrix showing proportion drivers in each Rail Mode across Shift Pattern and levels of Schedule Irregularity.

Nearly all passenger drivers (95%) reported working 8 h shifts, compared to 26% of freight drivers. One third of freight drivers reported working 12 h shifts, with 44% working shifts of varied lengths. Drivers rated their schedules as regular (19%), sometimes regular (22%) irregular (23%), or very irregular (36%). Schedules for freight drivers were more irregular than for passenger drivers (Fig. 1). The majority of drivers were on a schedule that included a combination of morning, evening, and night shifts. Three quarters of freight drivers and half of passenger drivers worked this combination. A larger number of passenger drivers were on permanent shift arrangements (Fig. 1).

### Sleep length and quality

**Figure 2:**
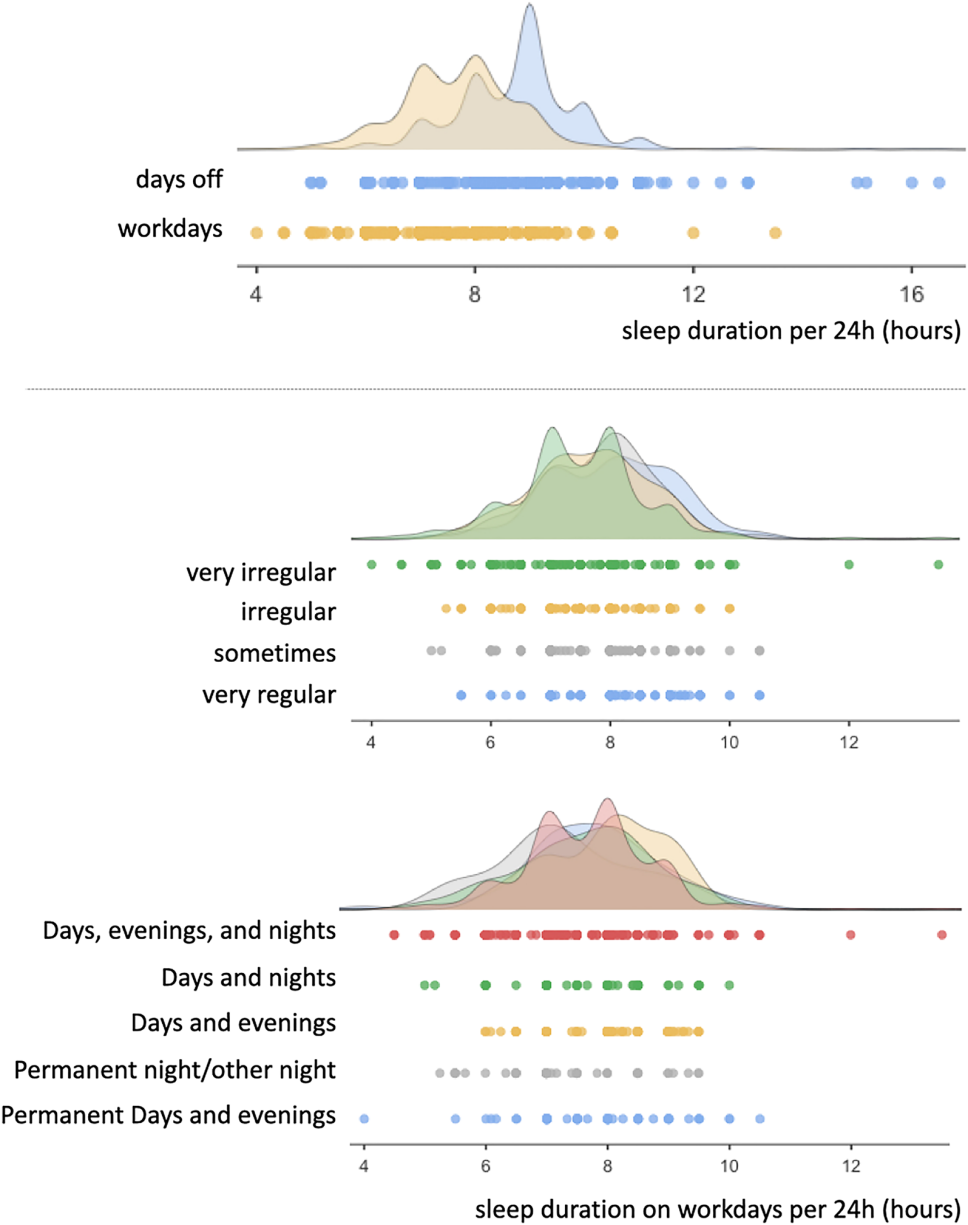
Adapted raincloud plots for sleep on days off compared to workdays (upper) and sleep on workdays split by schedule regularity and by shift pattern (lower).

On average, drivers reported fewer hours of sleep per 24 h on workdays compared to days off (Fig. 2), upper panel), with an average of 7.7 (± 1.1) hours workdays, compared to 8.7 (± 1.2) hours on days off. This 1 h difference in sleep duration was significant ($t_{731}=24.3, p<0.001$). Drivers reported lower sleep quality on workdays (6.2 ± 1.7) compared to days off (7.4 ± 1.4, $t_{747}=22.0, p<0.001$), representing a one-point difference on the 10-point scale.

Results of ANCOVA examining differences in sleep duration and quality on workdays and days off are displayed in Table 1. Older age was associated with lower sleep quality ratings on workdays ($p=0.001$), and lower sleep duration on days off ($p<0.001$). Males reported significantly lower sleep duration on days off ($p=0.003$). Longer shift lengths were associated with lower sleep quality ratings on workdays and days off ($p<0.05$).

Increasing schedule irregularity was associated with workday sleep durations that were shorter on average, and also more variable (Fig. 2, lower panel). Controlling for age, gender, rail mode, shift length, and days worked per week, there was a significant effect of regularity on workday sleep duration ($p<0.001$), such that on average, those on very regular schedules reported significantly higher amounts of sleep than those on irregular, or very irregular schedules ($p<0.05$). Controlling for the contribution of other factors, the only predictor with a large effect size for the relationship with sleep on workdays was schedule regularity ($\eta ^2$ = 0.349).

Shift pattern was also associated with workday sleep durations, such that durations were more variable on patterns that consisted of a mixture of days, evenings, and night shifts (Fig. 2, lower panel). Those who worked day and/or evening shifts reported significantly longer sleep duration than those on permanent nights or other shift schedules including nights ($p=0.049$).

**Table 1:** ANCOVA for sleep duration and quality on workdays and days off, controlling for age, gender, and number of days per week worked.

	Sleep duration						Sleep quality					
	Work			Off			Work			Off		
Predictor$_{num.df}$	$F_{730}$	*p*	$\eta ^{2}$	$F_{730}$	*p*	$\eta ^{2}$	$F_{730}$	*p*	$\eta ^{2}$	$F_{730}$	*p*	$\eta ^{2}$
Age$_{1}$	$<0.1$	0.916	$<0.001$	10.6	0.001	0.015	12.3	0.001	0.017	0.8	0.361	0.001
Gender$_{1}$	$<0.1$	0.876	$<0.001$	8.8	0.003	0.012	$<0.1$	0.856	$<0.001$	3.1	0.080	0.004
Days per week$_{1}$	3.1	0.077	0.004	0.3	0.578	$<0.001$	0.5	0.484	$<0.001$	1.1	0.293	0.002
Shift length$_{1}$	0.7	0.413	$<0.001$	0.1	0.754	$<0.001$	4.9	0.027	0.007	6.5	0.011	0.009
Mode$_{1}$	0.1	0.768	$<0.001$	0.2	0.676	$<0.001$	3.3	0.072	0.004	0.6	0.455	0.001
Regularity$_{3}$	8.7	$<0.001$	0.349	3.1	0.025	0.013	13.5	$<0.001$	0.052	1.1	0.334	0.005
Shift pattern$_{4}$	2.4	0.049	0.013	0.8	0.525	0.004	0.2	0.951	$<0.001$	1.1	0.351	0.006

Post-hocs for gender—days off sleep (F > M), Post-hocs for regularity (regular = 1, somewhat = 2, irregular = 3, very irregular = 4)—workday sleep (1 > 3, 4), day off sleep (4 > 3), workday sleep quality (1, 2, 3 > 4); post-hocs for shift pattern (permanent day or evening = 1, permanent night or other including night = 2, day and evening = 3, day and night = 4, day, evening, and night = 5)—workday sleep (1, 3 > 2); $\eta ^{2}$ effect size—small = 0.01, medium = 0.06, large = 0.14^38^.

### Satisfaction with sleep amount, tiredness, and wellbeing

**Figure 3:**
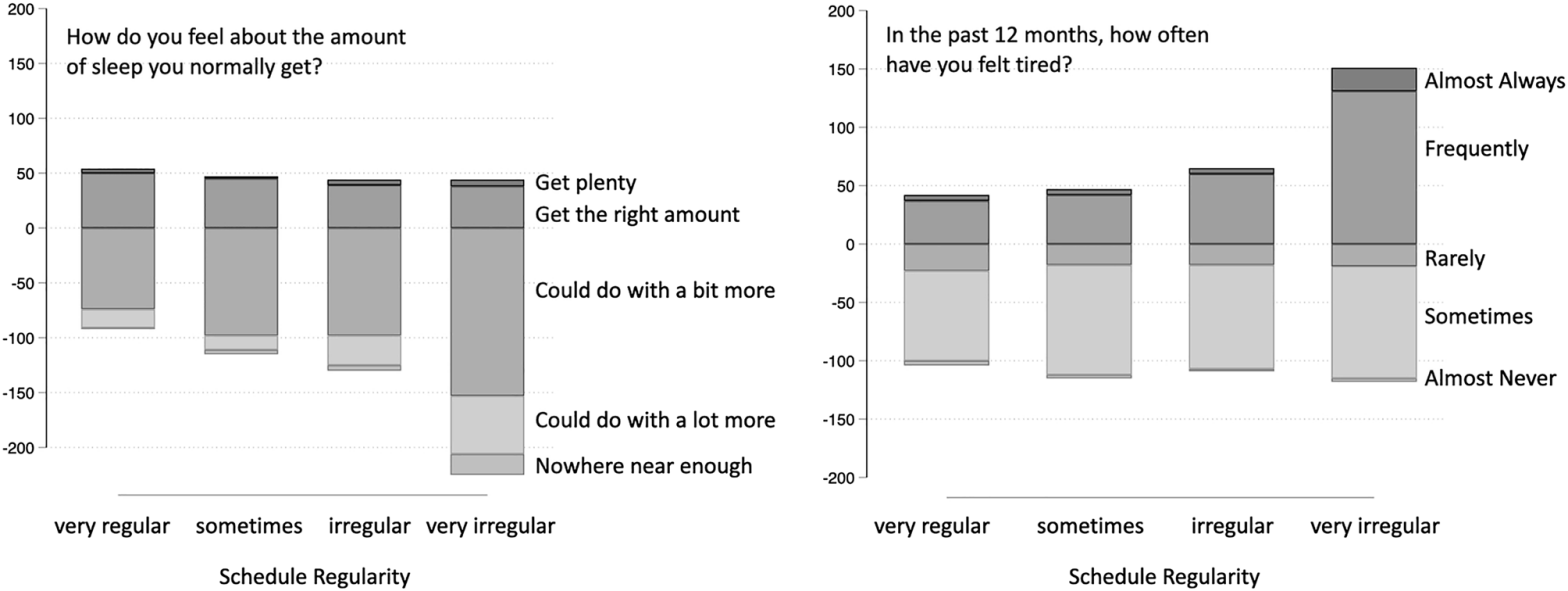
Slide plots for perceptions of sleep amount (left) and tiredness (right) split by schedule regularity (lower).

Approximately one in five drivers reported that they got plenty, or the right amount of sleep. The majority of drivers reported that they “could do with a bit more” sleep (56%), nearly one in four reported that they “could do with a lot more” (23%), and 2% reported that they got “nowhere near enough.” Just over one in ten drivers reported that they almost never or rarely felt tired (12%). Nearly half of drivers reported that they felt tired sometimes (47%), 36% reported that they felt tired frequently, and 5% that they almost always felt tired (Fig. 3).

Results of ordinal regression to investigate differences in satisfaction with sleep amount and frequency of tiredness are shown in Table 2. Age was a significant predictor of sleep amount satisfaction ($p=0.034$) and tiredness ($p<0.001$), such that older drivers formed a greater proportion of those who reported higher ratings of sleep satisfaction and lower ratings of tiredness. These effects were small (changes in odds for every year of age were 1–2%). There were no sex differences in sleep satisfaction, but there were for tiredness ($p=0.003$), such that females accounted for a higher proportion of those reporting higher tiredness. The effect was small, representing a 3% change in odds. Increasing shift length was associated with decreasing satisfaction with sleep amount ($p=0.001$), with a 1-h increase in shift length accounting for a 26% change in odds.

Controlling for the other factors, decreasing schedule regularity was significantly associated with decreased sleep amount satisfaction and increased tiredness (Fig. 3). These effects of schedule regularity were the largest effects in the models, resulting in a 69% change in odds across sleep satisfaction, and a 117% change in odds across tiredness for very irregular compared to regular schedules. There were no significant effects of shift pattern.

**Table 2:** Ordinal regression for perception of sleep satisfaction (1 = nowhere near enough) and tiredness (1 = almost never) controlling for age, gender, and number of days per week worked.

Variable (ref level)	Level	Sleep satisfaction				Tiredness			
		OR	Sterr	*z*	*p*	OR	Sterr	*z*	*p*
Age		1.01	0.01	2.1	0.034	0.98	0.01	$-$ 2.9	0.004
Gender (male)	Female	0.75	0.17	$-$ 1.3	0.200	1.97	0.44	3.0	0.003
Days per week		0.98	0.07	$-$ 0.3	0.764	0.89	0.06	$-$ 1.7	0.088
Shift length		0.74	0.07	$-$ 3.3	0.001	1.10	0.10	1.1	0.252
Mode (freight)	Passenger	0.88	0.18	$-$ 0.6	0.530	0.88	0.18	$-$ 0.6	0.529
Regularity (regular)	Sometimes	0.83	0.19	$-$ 0.8	0.406	0.76	0.18	$-$ 1.2	0.236
	Irregular	0.57	0.13	$-$ 2.4	0.015	1.01	0.23	0.1	0.956
	Very irregular	0.31	0.07	$-$ 5.1	$<0.001$	2.17	0.49	3.5	$<0.001$
Shift pattern (perm. days or evenings)	Perm. or other night	1.09	0.42	0.2	0.818	1.71	0.64	1.4	0.154
	Day and evening	1.08	0.33	0.3	0.787	1.20	0.36	0.6	0.555
	Day and night	1.03	0.37	0.1	0.923	1.61	0.57	1.3	0.181
	Day, evening, night	1.10	0.29	0.3	0.733	1.50	0.40	1.5	0.132

Post-hocs for regularity (regular = 1, somewhat = 2, irregular = 3, very irregular = 4)—sleep satisfaction (1 > 2 > 3 > 4), tiredness (1, 2, 3 < 4); *perm.* permanent.

**Figure 4:**
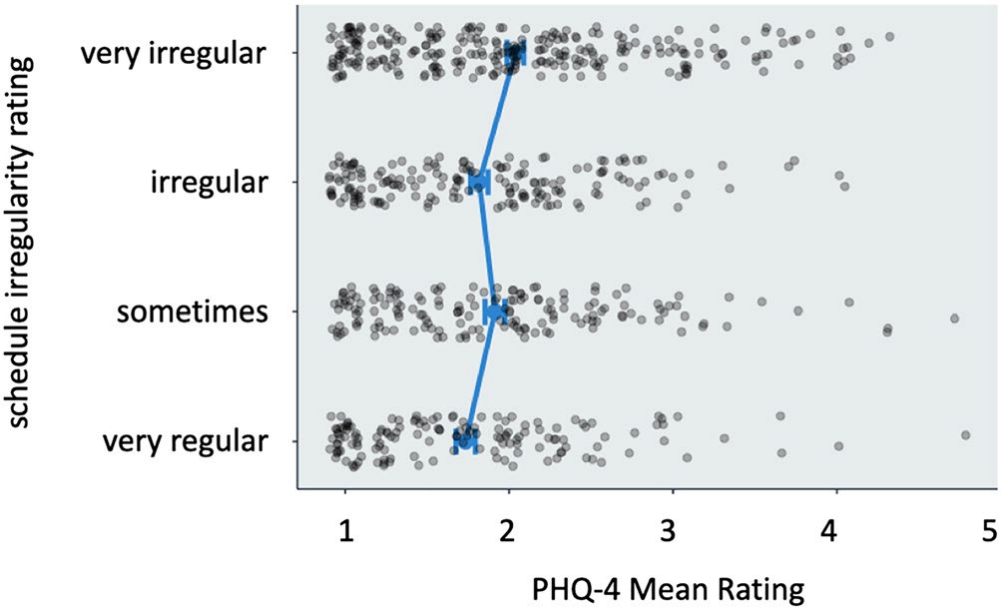
PHQ-4 ratings split by schedule irregularity.

Average score on the 5-point PHQ scale (1 = never experiencing symptoms of reduced psychological wellbeing, 5 = always) was 1.9 (± 0.8). Results of ANCOVA indicated that scores decreased (improved) with age ($F_{1,730} = 20.2, p<0.001, \eta ^2=0.027$) and that females reported significantly higher (worse) scores than males ($F_{1,730}=11.9, p<0.001, \eta ^2=0.016$). There was a significant effect of schedule regularity ($F_{3,730}=4.8, p=0.003, \eta ^2=0.019$), such that those on very irregular schedules had significantly higher (worse) scores than those on irregular, or very regular schedules. The effects for days per week ($F_{1,730}=11.9, p<0.001, \eta ^2=0.027$), shift length ($F_{1,730}=0.4, p=0.511, \eta ^2=0.001$), rail mode, ($F_{1,730}=0.4, p=0.554, \eta ^2<0.001$), and shift pattern ($F_{4,730}=0.8, p=0.537, \eta ^2=0.004$) were not significant. Mean values were near 2 (Fig. 4), and all of the effects ($\eta ^2$) are small at most.

### Impact of fatigue, health and wellbeing factors on driving performance

**Figure 5:**
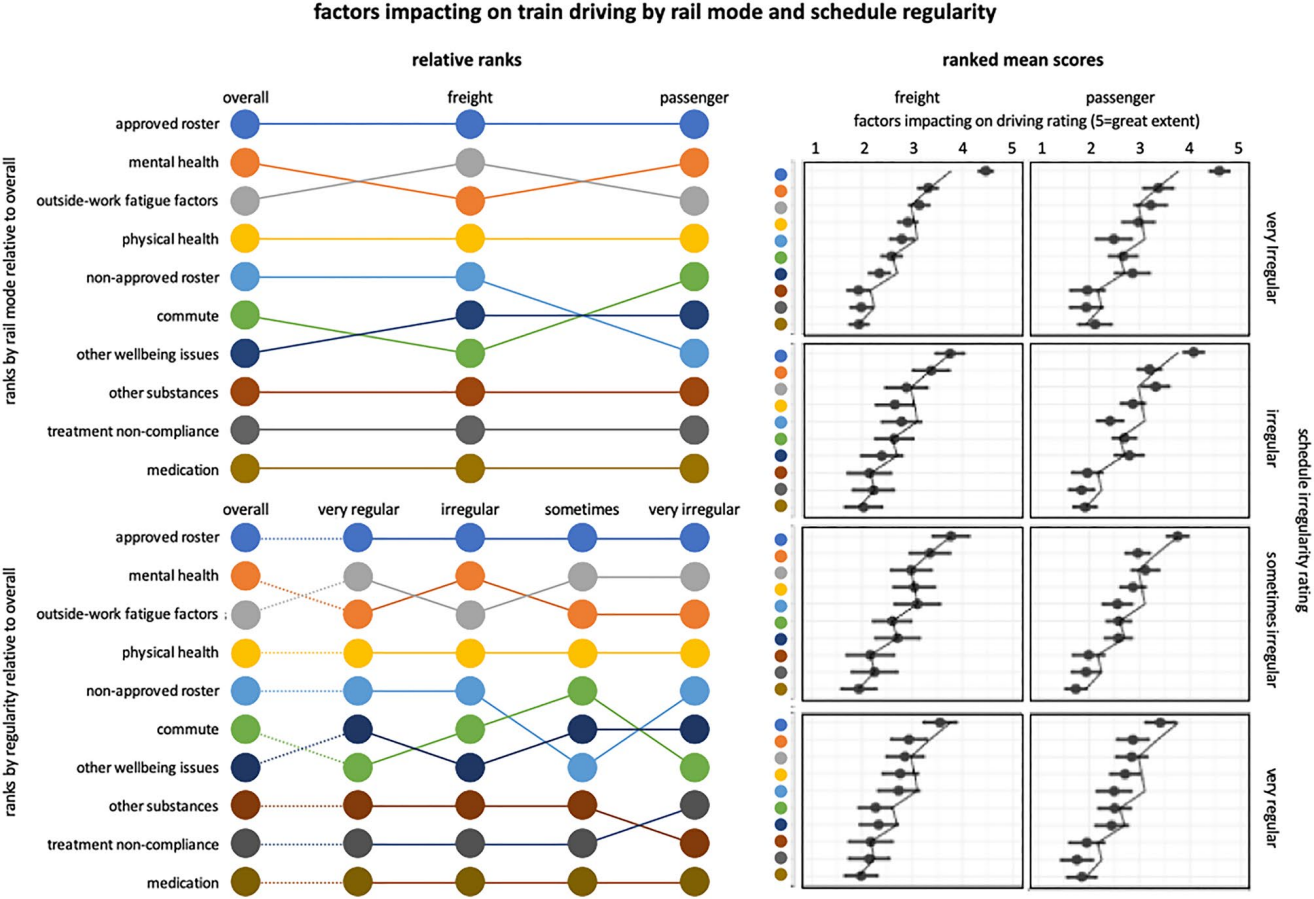
Bump Chart (left) displays ranking (according to mean rating on each scale, top = highest) of each of the fatigue, health, and wellbeing factors impacting on driving performance shown overall, and split by driving mode (upper) and regularity (lower). Plot matrix (right) display means (whiskers = sem) for each of the factors, ranked by mean rating, split by schedule regularity (rows), and rail mode (columns).

Of the ten factors, drivers ranked their approved roster as the factor with the strongest negative impact on their day-to-day driving performance, with a mean of 4.1 (± 1.1) on the 5-point scale (where 5 = driving is impacted to a great extent). Second was mental health (3.1 ± 1.4), followed closely by outside work fatigue factors (3.1 ± 1.3), and physical health (2.8 ± 1.3). Ratings for the other factors ranged from 2.6 ± 1.3 to 1.9 ± 1.2. Overall rankings for each of the ten factors are shown in the bump plots in Fig. 5. The rankings reflect the mean ratings, shown for freight and passenger modes (columns) and for schedule regularity (rows) on the right of the figure. Rankings for freight and passenger drivers were similar, outside work fatigue factors and non-approved roster factors featuring higher in the rankings for freight drivers, and mental health and commute factors featuring higher in the rankings for passenger drivers. The lower panel shows that rankings across different rated levels of schedule regularity are also relatively consistent. For those on very irregular schedules, non-approved roster factors featured more highly in their ranking, and the commute was lower on the ranking.

Results of MANCOVA investigating differences across all ten factors gender (Model $F_{12,5618.2}=3.11, p<0.001, \lambda =0.61$), indicated that there were significant overall effects of age ($F_{1,743}=2.5, p=0.007, \lambda =0.97$), mode ($F_{1,743}=3.3, p=0.002, \lambda =0.96$), and regularity ($F_{3,743}=4.8, p<0.001, \lambda =0.82$), which had the largest effect, explaining 18% of the variance. The effects for gender ($F_{1,743}=1.6, p=0.108, \lambda =0.98$), days per week ($F_{1,743}=1.4, p=0.187, \lambda =0.98$), shift length ($F_{1,743}=1.3, p=0.233, \lambda =0.98$), and shift pattern ($F_{4,743}=1.2, p=0.159, \lambda =0.94$) were not significant.

Post-hoc models for the effects of rail mode and schedule regularity are shown in Table 3. Ratings for the impact of the commute on driving performance were significantly higher ($p=0.021$) for passenger drivers (mean = 1.91 ± 0.79) than for freight drivers (mean = 1.89 ± 0.76). This was a small effect ($\eta ^2$ = 0.007). There were significant effects of regularity for the impact of physical health ($p=0.047$), mental health ($p=0.003$), approved roster ($p<0.001$), and outside work factors ($p<0.001$) on driving performance. The largest effect was for approved roster, representing a medium effect size ($\eta ^2$ = 0.132). Those on very irregular schedules reported significantly higher ratings than those on regular schedules ($p<0.05$) for the impact of mental health and outside work factors on driving performance. Those with more irregular schedules gave higher ratings for the impact of approved roster on driving performance, with significant differences between all levels of schedule regularity.

**Table 3:** MANCOVA post-hoc investigation of rail mode and regularity, controlling for age, gender, days per week, shift length, and shift pattern.

Factors affecting driving	Rail mode			Regularity		
	$F_{1,743}$	*p*	$\eta _{2}$	$F_{3,743}$	*p*	$\eta _{2}$
Physical health	0.8	0.371	0.001	2.7	0.047	0.011
Mental health	2.5	0.111	0.003	4.1	0.007	0.016
Medication	0.3	0.614	< 0.001	0.9	0.431	0.004
Other substances	0.4	0.538	< 0.001	0.2	0.907	< 0.001
Treatment non-compliance	1.8	0.180	0.002	0.4	0.792	0.001
Approved roster	3.1	0.078	0.004	37.0	< 0.001	0.132
Not approved roster	0.8	0.363	0.001	0.2	0.929	< 0.001
Commute	5.4	0.021	0.007	0.8	0.519	0.003
Outside work factors	1.1	0.304	0.001	4.3	0.005	0.017
Other wellbeing issues	0.4	0.547	< 0.001	1.5	0.202	0.006

Comparisons for mode (freight = f, passenger = p)—commute (p > f); Comparisons for regularity (regular = 1, somewhat = 2, irregular = 3, very irregular = 4)—physical health (4 > 1), mental health (4 > 1), approved roster (4 > 3 > 2 > 1), outside work factors (4 > 1).

## Discussion

Results from this large survey of train drivers from Australia and New Zealand reinforce previous research indicating that fatigue is a workplace hazard in rail^39^, as in other industries^40,41^. One in four drivers in the survey reported that they could do with a lot more sleep, or got nowhere near enough sleep and approximately 40% reported that they frequently or almost always felt tired. With train driving requiring a high degree of attention and concentration^2,3,6^, and a well-established relationship between fatigue and ability to maintain attention^11,15,16^, these results emphasize the extent of the threat posed by fatigue in the rail sector. In examining the relationship between schedules, sleep, wellbeing, and fatigue, a key factor that emerged, with the largest and most consistent negative effects (and controlling for other factors) was schedule irregularity. While drivers perceived the roster as the most important factor impacting on their driving performance, mental health, physical health, and outside work factors were also among the highest ranks.

The results highlight the role of rostering in generating fatigue, and in particular the perceived impact on driving performance. Consistent with other research, sleep durations were more variable for shift patterns that consisted of a mixture of shifts (days, evenings, nights), and longer hours were associated with lower sleep quality ratings and a reduction in satisfaction with sleep amount^10,42^. Approved rosters were ranked as having the most important impact on day-to-day driving performance, and this was consistent across rail mode (freight and passenger drivers), and categories of identified schedule irregularity. Following approved roster, the highest-ranked perceived impacts on driving performance came from physical and mental health, and outside work factors. Research has identified reciprocal relationships between work factors (e.g. roster, task characteristics such as sedentary work), non-work factors (domestic/caring responsibilities, level of physical activity, sleep, physical health, mental health), and performance and safety in shiftworkers, including train drivers^22,24,26,30,43–47^. This is reflected in driver perceptions in this study, and reinforces, not only the reciprocal employee-employer duty of care^48,49^, but also the need to consider train drivers within interacting inside- and outside- work systems^6,41,50^, in order to appropriately approach fatigue management.

The results also highlight the impact of irregularity in rostering. Schedules are typically referred to as irregular if the hours (amount and/or timing) are variable over a roster period (e.g., a week, a month), and irregularity is often used in tandem with concepts of predictability, insufficient notice of work, and “on-call” work, connected in the literature with poorer sleep, performance, health, and safety^51–56^. Irregular work is generally considered to present particular challenges for fatigue risk management through, as an example, difficulties in estimating likely sleep amounts and related fatigue^53–55^. In this study, schedule irregularity was associated with shorter and more variable sleep duration, and also had the biggest effect size for workday sleep duration, larger than age, gender, number of days worked per week, shift length, and shift pattern. The results also suggested that schedule irregularity may amplify the negative impacts of the roster, impaired physical and mental health, and outside work factors on driving performance (as indicated by the strength of the effect size for irregularity in the model). In this study, irregularity was measured using a single, four-point, ordinal scale, with no definitions or descriptions provided for the participant (unlike the Standard Shiftwork Index^34^, which provides descriptions). A notable limitation of this study is the limited way in which irregularity was captured, which did not include more specific schedule information such as variation in time-off between shifts (e.g., caused by short shift intervals^57^, sometimes referred to as ‘quick returns’). This would have provided more context to responses to our subjective questions. Indeed, given the overlap in the literature and guidelines between irregularity and related concepts such as predictability, notice, and “on-call,” future research investigating the concept of schedule irregularity would be highly beneficial. Importantly, qualitative research to delve into how train drivers understand irregularity in their work context, with work towards identification of key concepts, definitions, and methods of measurement would be of great value. Combining these methods with more objective measurements, such as operational roster and payroll records, would provide critical converging evidence, as such studies have identified relationships between roster irregularity, short shift intervals, and absences due to illness^57,58^.

Alongside the negative impacts arising from the schedule, there was evidence that train drivers engaged in compensatory behaviours, including sleeping for longer on days off. Consistent with previous research, (e.g., Refs.^10,46,59^) train drivers averaged approximately one hour less sleep on workdays compared to days off. Sleep was also reportedly of higher quality on days off compared to workdays. In comparison, the effects of factors related to the individual, such as gender and age, had relatively small and more mixed effects. For gender, females accounted for a higher proportion of those reporting higher tiredness and lower psychological wellbeing, and males reported lower sleep duration on days off. Sex differences in sleep have been identified in the literature, perhaps most consistently explained by differences in the circadian system^60^. Whether there is an objective difference in sleep per se, as opposed to reflections of circadian differences in addition to differences in reporting, is less clear^61^. While increasing age was associated with lower sleep duration on days off, and reduced sleep quality on workdays, older drivers reported lower tiredness, higher sleep satisfaction, and increased psychological wellbeing. This is consistent with research showing that while there are sleep changes associated with ageing, they are not consistent, nor are they clearly related to deficits in wellbeing or performance^46,62,63^. An important consideration in relation to interpreting the age effects is the possible influence of the “healthy survivor effect” for shiftwork^46,64,65^. This is the phenomenon whereby, as shiftworkers age, those who are more vulnerable to the negative impacts of shiftwork may, if they are able, choose to reduce their exposure to shiftwork, either partially, or entirely. This results in an older shiftworker cohort who may cope with shiftwork particularly well, and who are therefore healthier and safer. This presents a limitation for the current study since the cross-sectional survey design only allows us to test (and control) for the current age of respondents. Without a longitudinal design, we are unable to separate the effects of aging and selection out of shiftwork. Studies that have done so have suggested that those who opt out of shiftwork have less healthy profiles than those who remain, the bifurcation becoming more apparent as age progresses (e.g., Ref.^65^).

Interestingly, there was little difference between freight and passenger drivers, with rankings relatively consistent across measures of fatigue, health, and wellbeing. However, rankings on the impact of the commute to and from work were significantly higher for passenger drivers, indicating that the commute might be a key factor impacting driving performance in urban environments. Commute impacts have been identified as important in shiftworking industries^66–69^, and are treated highly inconsistently across workplaces and industries in fatigue risk management considerations, with some guidelines using ’door-to-door’ scheduling (i.e., including travel time from and back to home in work hours).

Rostering principles to manage fatigue risk are available for application in rail operations (e.g. Ref.^41^) but these focus on specific areas such as shift length, breaks between shifts, numbers of consecutive shifts and rotation of shift patterns. This research supported existing good roster practices, however, the strongest influences identified in this study were shift variability and schedule irregularity, and these concepts are often poorly represented in existing industry guidance. Drivers also identified physical and mental health alongside roster practices as strong contributors to their driving performance. These results provide evidence for reflection on current fatigue management guidelines for train drivers, suggesting a focus on schedule irregularity, and underscoring the importance of a systems approach^16,41,48,50^, recognising the multiple, interrelated factors that impact on performance and safety.
